# Supplementary material for: Chromothripsis during telomere crisis is independent of NHEJ, and consistent with a replicative origin
Source: Genome Res. 2019 May;29(5):737–49. doi: 10.1101/gr.240705.118 (PMC6499312; doi:10.1101/gr.240705.118)
Supplement: Supplemental Material [file supp_gr.240705.118_Supplemental_file_1.zip › contigs/annotated_contigs/DB55/contig.2.DB55_length_1092_mean_cov_8.36538461538.docx]

**DB55_length_1092_mean_cov_8.36538461538**

```
TCAATCCATCACTCAACATTGAATGCTTCCTATATGCCTGGCACTCTGTTGAGCTCTGGGAATGAAAAGGTGAGCAGAGGATTATATTA
 >chr7:144870595-144870963 + E=3e-209
GAAACATAATTCAATTTACTGTCATACCCAACATCTCTCCCAGTTTAACAGCTTCTCCTTTAGTTCTTGGAAAATACAGGCCCCTGTTC

CCTTTGGCACACTCTCATTCCCAAGTCACAACCAAATGGGTCAGGAATGTGCACCTGCCCTGACACCAGGCAATCAATTGACGAGATGC

TCTCTCTTCAGAATTTGAAGATGGTGACATTATGGCTGTAGTCAGTAAATTGCTGAGTGTAAGTGTATGAGTTATGACTTTATATCCAA

GTGATGGTC|ACA|CCTGAAATTTTATCATGAGCCAGATGCTGAGGAAGAGAGAAAAAGCACAATCATCATTGAAATTAGAGCTTCTGG
          >chrUn_gl000218:117437-118142 - E=0e+00
CTGGGCGCGGTGGCTCACGCCTGTAATCCCAGCACTCTGGAAGGCCGAGGTGGGCGGATCACAAGGTCAGGAGATCGAGACCATCCTGG

CTAACACGGCAAAACCCCGTCTCTACTAAAAATACAAAAAATTAGCTGGGTGAGGTGGCGGGCGCCTATAGTCCCAGCTACTCAGGAGG

CTGAGGCAGGAGAATGGCGTGAATCCAGGAGGCAAAACTTCAGGTTTTGCCAAGAATGACACTGTAAATGTAACAAAGCTTCTGTGCTT

GTTAGTGAACACCAAATCAGCTACTCTCCTGTATTCGGAGATCAGGATGAAATGAAAAGAACCAGCAGGCCGGGCACGGTGGCTCATGC

CTGTAATGTCAACACTTTGGGAGGCTTAGGTGTGCGGATCACCCGAGGTTGGGAGTTCGAGACCAGCCTGACCAACATGGAGAAACCCC

GTCTCTACTAAAAATGCAAAATGAGCTGGGCGTGGTGGCACAAGCCTGTAATCCCAGCTTGGGAGGCTGAGGCAGGATAAGTGCTTAAA

CCTGAGAGGCGGAGGTTGCTGTGAGGCAATATTGCACCATTGCACTCCAGCCTGGGCAACAAGAGTGAAACTCCATCTCAAAAAAAAAA

AAAA|AAATAAAAAGCCCCCCACCCTT
```
